# Propensity score-adjusted analysis on stent-assisted coiling versus coiling alone for ruptured intracranial aneurysms

**DOI:** 10.1038/s41598-021-01156-y

**Published:** 2021-11-05

**Authors:** Lukas Goertz, Thomas Liebig, Lenhard Pennig, Marco Timmer, Hanna Styczen, Jan-Peter Grunz, Thorsten Lichtenstein, Marc Schlamann, Christoph Kabbasch

**Affiliations:** 1 Center for Neurosurgery, Medical Faculty and University Hospital, University of Cologne, Kerpener Strasse 62, 50937 Cologne, Germany; 2 Department of Neuroradiology, Medical Faculty and University Hospital, University of Cologne, Kerpener Strasse 62, 50937 Cologne, Germany; 3 Department of Neuroradiology, LMU University Hospital of Munich, Marchioninistraße 15, 81377 Munich, Germany; 4 Institute for Diagnostic and Interventional Radiology and Neuroradiology, University Hospital Essen, Hufelandstraße 55, 45147 Essen, Germany; 5 Department of Diagnostic and Interventional Radiology, University Hospital Wuerzburg, Josef-Schneider-Straße 2, 97080 Wuerzburg, Germany

**Keywords:** Neurological disorders, Stroke

## Abstract

Stent-assisted coiling (SAC) for ruptured intracranial aneurysms (RIAs) remains controversial due to an inherent risk of potential thromboembolic and hemorrhagic complications. We compared SAC and coiling alone for the management of RIAs using propensity score-adjustment. Sixty-four patients treated by SAC and 220 by stand-alone coiling were retrospectively reviewed and compared using inverse probability of treatment weighting (IPTW) with propensity scores. Functional outcome, procedure-related and overall complications and angiographic results were analyzed. Aneurysms treated by SAC had a larger diameter, a wider neck and were more frequently located at the posterior circulation. SAC had a higher risk for thromboembolic complications (17.2% vs. 7.7%, p = 0.025), however, this difference did not persist in the IPTW analysis (OR 1.2, 95% CI 0.7–2.3, adjusted p = 0.458). In the adjusted analysis, rates of procedural cerebral infarction (p = 0.188), ventriculostomy-related hemorrhage (p = 0.584), in-hospital mortality (p = 0.786) and 6-month favorable functional outcome (p = 0.471) were not significantly different between the two groups. SAC yielded a higher complete occlusion (80.0% vs. 67.2%, OR 3.2, 95% CI 1.9–5.4, p < 0.001) and a lower recanalization rate (17.5% vs. 26.1%, OR 0.3, 95% CI 0.2–0.6, p < 0.001) than stand-alone coiling at 6-month follow-up. In conclusion, SAC of large and wide-necked RIAs provided higher aneurysm occlusion and similar clinical outcome, when compared to stand-alone coiling.

## Introduction

Endovascular coil embolization represents the standard treatment modality for intracranial aneurysms. The adjunct use of intracranial stents prevents coil protrusion into the parent artery and facilitates coiling of wide-necked aneurysms with an unfavourable dome-to-neck ratio^1,2^. Since stent-assisted coiling (SAC) allows for a denser coil packing, it provides better long-term aneurysm occlusion than balloon-assisted coiling or coiling alone^3,4^. Procedural complications of SAC are mainly attributable to thromboembolic and hemorrhagic events because of the mandatory anti-platelet therapy^5^. Particularly for the latter reason, the use of SAC for acutely ruptured aneurysms remains controversial. There is concern that double anti-platelet medication might increase the risk of aneurysm rebleeding and can complicate intracranial surgical procedures which might be necessary during the acute phase of subarachnoid hemorrhage (SAH)^6,7^. On the contrary, insufficient anti-platelet therapy may increase the risk of cerebral infarction, which is a major cause for treatment-related morbidity. Nevertheless, previous studies have provided evidence that SAC combined with state-of-the-art antiplatelet therapy can be safe and effective for the management of acutely ruptured aneurysms^8–10^. Moreover, a recent meta-analysis by Zhang et al. reported similar functional outcome and a lower recurrence rate for SAC when compared to stand-alone coiling^11^. However, since SAC is generally used for more complex aneurysms, a direct comparison between the treatment modalities is impeded. To date, there have been no prospective randomized studies comparing SAC to coiling alone and no comparative studies that systematically adjust for diverging baseline patient and aneurysm characteristics.

The objective of the current study was to compare SAC and coiling alone for ruptured intracranial aneurysms regarding procedural complications, clinical outcome, and angiographic results. In order to create homogenous study groups, retrospective randomization was performed using inverse probability treatment weighting (IPTW) based on individual propensity scores.

## Results

### Patient and aneurysm characteristics

**Figure 1:**
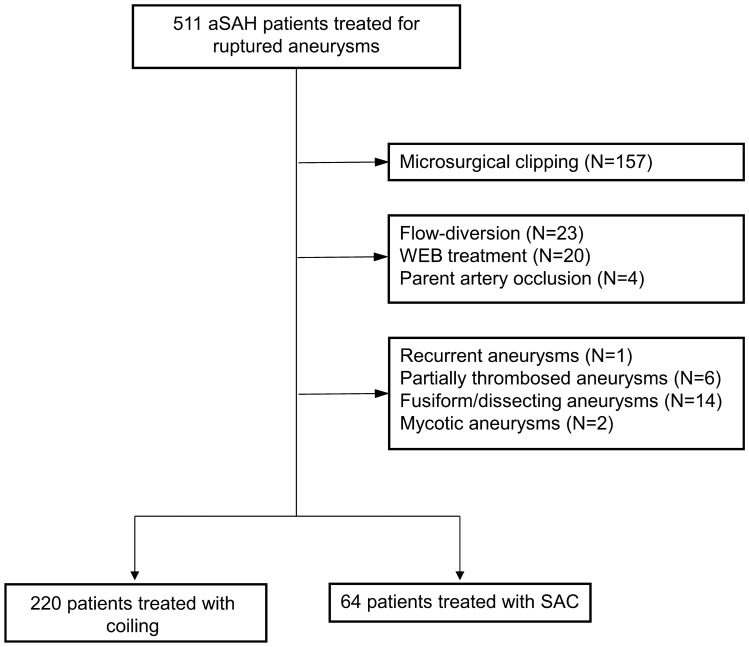
Flow chart of patient selection. aSAH, aneurysmal subarachnoid hemorrhage; WEB, Woven Endobridge; SAC, stent-assisted coiling.

During the study period, 511 patients were treated for a ruptured aneurysm. Thereof, 284 patients met the inclusion criteria and were enrolled. Patient selection is detailed in Fig. 1. The mean patient age was 54.3 ± 14.0 years and 183 patients were female (64.4%). The ruptured aneurysm was located at the intradural internal cerebral artery in 70 cases (24.6%), at the anterior cerebral artery in 127 (44.7%), at the middle cerebral artery in 31 (10.9%) and at the posterior circulation in 56 (19.7%). The mean aneurysm size was 7.5 ± 3.7 mm and the mean neck width was 3.3 ± 1.6 mm. The aneurysms were treated within 48 h after ictus in 242 cases (85.2%). Stand-alone coiling was performed in 220 patients (77.5%), while adjunctive stents were used in 64 (22.5%). In the coiling alone group, 30 patients were treated with balloon-assistance (13.6%). In the SAC group, stent implantation was planned a priori in 53 patients (82.8%) due to a wide aneurysm neck. In 11 patients (17.2%), the stent was implanted as a salvage technique, because the coils protruded or tended to protrude into the parent artery (n = 10), or to allow a denser coil packing (n = 1). Aneurysms treated with the assistance of intracranial stents were more frequently located at the posterior circulation (39.1% vs. 14.1%, p < 0.001), had a larger mean diameter (8.7 ± 4.4 mm vs. 7.2 ± 3.4 mm, p = 0.003) and a wider neck (4.5 ± 2.2 mm vs. 3.0 ± 1.2 mm, p < 0.001) than aneurysms treated without stent assistance. After IPTW, the two groups were comparable regarding all baseline characteristics as detailed in Table 1.

**Table 1:** Baseline patient and aneurysm characteristics.

	Unadjusted analysis			IPTW analysis		
	Coiling (n = 220)	SAC (n = 64)	P	Coiling (n = 287)	SAC (n = 251)	P
Patient age (years)	54.0 ± 14.4	55.5 ± 12.6	0.441	53.7 ± 14.4	54.2 ± 10.9	0.669
Gender			0.158			0.530
Female	137 (62.3%)	46 (71.9%)		187 (65.2%)	157 (62.5%)	
Male	83 (37.7%)	18 (28.1%)		100 (34.8%)	94 (37.5%)	
WFNS grade			0.847			0.231
WFNS 1	72 (32.7%)	20 (31.3%)		90 (31.4%)	80 (31.9%)	
WFNS 2	42 (19.1%)	9 (14.1%)		48 (16.7%)	28 (11.2%)	
WFNS 3	18 (8.2%)	5 (7.8%)		25 (8.7%)	17 (6.8%)	
WFNS 4	27 (12.3%)	10 (15.6%)		40 (13.9%)	35 (13.9%)	
WFNS 5	61 (27.7%)	20 (31.3%)		84 (29.3%)	91 (36.3%)	
Fisher grade			0.359			0.262
Fisher 1	2 (0.9%)	1 (1.6%)		3 (1.0%)	6 (2.4%)	
Fisher 2	15 (6.8%)	5 (7.8%)		21 (7.3%)	21 (8.4%)	
Fisher 3	103 (46.8%)	22 (34.4%)		124 (43.2%)	91 (36.3%)	
Fisher 4	100 (45.5%)	36 (56.3%)		138 (48.1%)	134 (53.4%)	
IVH	82 (37.3%)	33 (51.6%)	0.040	118 (41.1%)	123 (49.0%)	0.073
ICH	39 (17.7%)	10 (15.6%)	0.695	47 (16.4%)	40 (15.9%)	0.874
Aneurysm location						
ICA	57 (25.9%)	13 (20.3%)	0.361	69 (24.0%)	49 (19.5%)	0.206
ACA	106 (48.2%)	21 (32.8%)	0.030	127 (44.3%)	128 (51.0%)	0.120
MCA	26 (11.8%)	5 (7.8%)	0.366	32 (11.1%)	23 (9.2%)	0.448
PC	31 (14.1%)	25 (39.1%)	< 0.001	59 (20.6%)	51 (20.3%)	0.945
Aneurysm size (mm)	7.2 ± 3.4	8.7 ± 4.4	0.003	7.7 ± 4.0	8.1 ± 3.9	0.315
Neck width (mm)	3.0 ± 1.2	4.5 ± 2.2	< 0.001	3.3 ± 1.6	3.7 ± 1.5	0.110
Dome-to-neck ratio	1.9 ± 0.7	1.8 ± 1.0	0.310	1.9 ± 0.7	1.8 ± 0.9	0.071
Aneurysm treatment < 48 h after ictus	184 (83.6%)	58 (90.6%)	0.166	237 (82.6%)	220 (87.6%)	0.101

IPTW, inverse probability of treatment weighting; SAC, stent-assisted coiling; WFNS, World Federation of Neurosurgical Societies grading scale; IVH, intraventricular haemorrhage; ICH, intracranial hemorrhage; ICA, internal carotid artery; ACA, anterior cerebral artery; MCA, middle cerebral artery; PC, posterior circulation.

### Immediate aneurysm occlusion

The control angiography scan after the procedure showed complete aneurysm occlusion, neck remnants and aneurysm remnants in 85.5%, 9.5% and 5.0% for coiling, respectively, and in 87.5%, 10.9% and 1.6% for SAC, respectively (p = 0.549). After propensity score adjustment, SAC was associated with higher odds for complete aneurysm occlusion (OR 1.9, 95% CI 1.1–3.2, p = 0.018) while stand-alone coiling carried a higher risk for aneurysm remnants (OR 2.7, 95% CI 1.1–7.0, p = 0.030).

### Complications

Complications are listed in detail in Table 2. The SAC group was associated with a longer hospital stay (29.0 ± 21.4 days) than the coil group (27.0 ± 15.9 days, p = 0.411, adjusted p < 0.001). Overall intraoperative events occurred more often during SAC than during coiling alone (21.9% vs. 11.4%, OR 2.2, 95% CI 1.1–4.5, p = 0.032). After PS adjustment, this difference did not remain statistically significant (OR 1.2, 95% CI 0.7–2.0, p = 0.458). In the subanalysis of complication types, SAC carried higher odds for intraoperative thromboembolic events in the unadjusted analysis (OR 2.5, 95% CI 1.1–5.6, p = 0.025) but not in the adjusted analysis (OR 1.2, 95% CI 0.7–2.3, p = 0.433). Intraoperative hemorrhagic event rates were similar in both groups. Procedure-related cerebral infarction were similar in the coiling (3.6%) and the SAC group (3.1%, p = 1.0, adjusted p = 0.188). There were two cerebral infarctions related to SAC. The first patient was treated by Y-stent-assisted coiling for an anterior communicating artery aneurysm. Stent deployment caused a thromboembolic occlusion of a M3 branch of the MCA. The embolus could be dissolved by tirofiban, however, postoperative CT showed a partial MCA infarction. The patient had motor dysphasia after the procedure (mRS 3). In the second case, the patient had cerebellar and mesencephalic infarction after Y-stent-assisted coiling of a basilar tip aneurysm, although no intraoperative thromboembolic events were observed. Intensive care treatment was finally discontinued in this patient (mRS 6). Overall ventriculostomy-related hemorrhage rates were 14.0% in the coiling group and 9.3% in the SAC group (p = 0.367, adjusted p = 0.584). One patient in the coiling group required surgical evacuation of the intracranial hemorrhage after VP shunt placement. In the IPTW analysis, SAC was associated with increased odds of vasospasm (OR 1.7, 95% CI 1.2–2.4, p = 0.002), however, overall ischemic stroke rates were similar (OR 1.2, 95% CI 0.9–1.8, p = 0.257).

**Table 2:** Procedure-related complications.

	Coiling (n = 220)	SAC (n = 64)	P	Adjusted P
Length of stay (days)	27.0 ± 15.9	29.0 ± 21.4	0.411	< 0.001
Overall intraoperative procedural complications	25 (11.4%)	14 (21.9%)	0.032	0.458
Thromboembolic events	17 (7.7%)	11 (17.2%)	0.025	0.433
Hemorrhagic events	9 (4.1%)	3 (4.7%)	0.736	0.813
Procedural cerebral infarction	8 (3.6%)	2 (3.1%)	1.0	0.188
Ventriculostomy-related hemorrhage				
EVD	20/156 (12.8%)	3/53 (5.7%)	0.205	0.212
VP-Shunt	3/51 (5.9%)	3/17 (17.6%)	0.160	0.483
Overall	22/157 (14.0%)	5/54 (9.3%)	0.367	0.584
Vasospasm	94 (42.7%)	28 (43.8%)	0.884	0.002
Overall ischemic stroke	64 (29.1%)	25 (39.1%)	0.130	0.257

SAC, stent-assisted coiling; EVD, external ventricular drain; VP, ventriculoperitoneal.

There was no statistical difference among in-hospital mortality rates between coiling (17.7%, 39/220) and SAC (26.6%, 17/64, p = 0.118, adjusted p = 0.786). At discharge, 44.5% of coiled patients had a favourable mRS score, compared to 40.6% in the SAC group (p = 0.578, adjusted p = 0.766). Among survivors, 6-month clinical follow-up was available for 75.7% (137/181) in the coiling group and for 89.3% (42/47) in the SAC group. At 6-month follow-up, favourable outcome was achieved by 55.5% in the coiling group and by 53.1% in the SAC group (p = 0.742, adjusted p = 0.471).

### Angiographic outcome

Angiographic follow-up was available for 119 patients (54.1%) in the coiling group and 40 patients (62.5%) in the SAC group. Complete occlusion, neck remnants and aneurysm remnants were observed in 67.2%, 11.8% and 21.0% after coiling, respectively, and in 80.0%, 10.0% and 10.0% after SAC, respectively (p = 0.361). After adjustment for the propensity scores, SAC had significantly higher odds for complete aneurysm occlusion (OR 3.2, 95% CI 1.9–5.4, p < 0.001). Recanalization occurred in 17.5% after SAC and 26.1% after coiling (p = 0.273). After adjustment, sole coiling was significantly associated with aneurysm recurrence (OR 3.1, 95% CI 1.7–5.4, p < 0.001). Retreatment rates were 22.7% for the coiling group and 17.5% for the SAC group (p = 0.474). This difference became significant after IPTW adjustment (OR 1.7, 95% CI 1.0–3.6, p = 0.049).

## Discussion

The results of the current study demonstrate that SAC provides superior immediate aneurysm occlusion to stand-alone coiling, when accounting for diverging baseline characteristics. SAC was associated with higher risks of periprocedural thromboembolic events, however, this effect was mitigated after propensity score adjustment. In this context, there were no significant differences among procedural cerebral infarction, overall ischemic stroke, in-hospital mortality, and ventriculostomy-related hemorrhage rates. A similar portion of patients in both groups achieved favourable functional outcome. SAC provided higher 6-month complete aneurysm occlusion rates, requiring retreatment less frequently than coiling alone.

Previous studies indicated that SAC carries a higher risk of thromboembolic complications than stand-alone coiling. For instance, Hetts et al. reported 1-year ischemic stroke rates of 8.8% for SAC compared to 2.2% for coiling^3^. In the current study, SAC had a relative risk of 2.5 for thromboembolic complications, hence supporting previous studies on mostly unruptured aneurysms. However, after propensity score adjustment, this difference was mitigated. In addition, procedure-related and overall cerebral infarction rates were comparable between both groups. Generally, SAC is performed predominantly for large, wide-necked and bifurcation aneurysms. A subanalysis of the aneurysms included in the CLARITY study demonstrated that these anatomical features represent per se an increased risk for thromboembolic complications^12^. However, the difference in baseline aneurysm characteristics between SAC and coiling alone is not statistically addressed by most comparative studies. Furthermore, prospective randomized studies on this topic are lacking. To our knowledge, this is the first study comparing conventional coiling and SAC using an IPTW approach, which simulates a retrospective randomization. Although IPTW cannot substitute a prospective randomized clinical trial, adjustment for the individual propensity scores allows a direct comparison of the two treatment modalities, which can be regarded as a strength of the present study. Our findings indicate that SAC has a higher risk of thromboembolic complications in clinical practice, however, this may be mainly related to the preferential treatment of morphologically complex aneurysms and not necessary by the procedure itself.

To identify a correlation between dual anti-platelet aggregation and intracranial bleeding tendency, we determined the frequency of intracranial hemorrhage after ventriculostomy. SAC patients carried a slightly higher risk of radiographic hemorrhage related to VP-shunt placement, however, this difference was not statistically significant and the hemorrhage did not require surgical evacuation in any case. In contrast, EVD-related haemorrhage occurred less frequently after SAC than after coiling, although this difference did not receive statistical significance either. Due to the necessary anti-platelet treatment related to SAC, one would expect increased ventriculostomy-related haemorrhage for these patients. Accordingly, Kung et al. revealed anti-platelet therapy as significant risk factor for both radiographic and symptomatic intracranial hemorrhage after ventriculostomy^13^. Darkwah-Oppong et al. confirmed an association between anti-platelets and ventriculostomy-induced bleedings, however it had no impact on functional outcome in their study^14^. Our institutional SAH protocol includes EVD placement before interventional aneurysm embolization and start of anti-aggregant therapy. This approach might increase the safety of ventriculostomy in case of subsequent stent implantation. Moreover, Darkwah-Oppong et al. reported that aspirin monotherapy was associated with lower odds for hemorrhagic complications than dual antiplatelet therapy^14^. Likewise, antiplatelet therapy did not affect functional outcome in a subanalysis of the International Subarachnoid Aneurysm Trial^15^. Nevertheless, most neurosurgeons would agree that restriction to aspirin monotherapy would be a considerable advancement for SAC of ruptured aneurysms. There are first reports on single antiplatelet therapy with coated flow-diverters^16,17^, however, a systematic clinical evaluation of its safety and efficacy is lacking so far and definite conclusion cannot be drawn yet.

In the current study, SAC carried increased odds of cerebral vasospasm. This result is opposed to previous findings. Andic et al. reported that SAC in the presence of vasospasm is feasible and safe and the stent can provide additional mechanical vasodilation^18^. Nagahama et al. reported a lower risk for vasospasm and delayed cerebral ischemia among patients receiving dual anti-platelet therapy^19^. Likewise, Darkwah-Oppong et al. reported a reduced risk of delayed cerebral ischemica and more favourable outcome among SAH patients under aspirin therapy^20^. In our study, the cerebral infarction rates were comparable between SAC and coiling and a similar portion of patients achieved favourable clinical outcome in both groups. These findings are in line with previous studies. Zhang et al. conducted a meta-analysis that included eight retrospective studies comparing SAC and stand-alone coiling of ruptured aneurysms. The authors reported increased rates of hemorrhagic (OR 1.6, 95% CI 1.1–2.4, p = 0.319) and thromboembolic events (OR 1.8, 95% CI 1.3–2.4, p = 0.511) in the SAC group, however, the favourable clinical outcome rates was similar in both groups (OR 0.95, 95% CI 0.88–1.02, p = 0.338). Summarizing these results, anti-platelet medication after SAC might slightly increase the risk of intracranial bleeding, however, these are mostly minor incidents associated with low morbidity.

Numerous studies have shown higher complete occlusion and lower recurrence rates of SAC compared to coiling without stent-assistance^3,4,21,22^. Jahshan et al. reported complete aneurysm occlusion in 64.6% after SAC compared to 49.7% after coiling alone^22^. Piotin et al. described aneurysm recanalization in 14.9% after SAC compared to 33.5% after sole coiling^4^. In the meta-analysis on ruptured aneurysms by Zhang et al., the immediate complete occlusion rate in the SAC groups was significantly lower than that in the non-SAC group (54.3% vs. 64.2%, OR 0.90, 95% CI 0.83–0.99)^11^. However, SAC achieved a mid-term occlusion rate of 73.4% compared to 61.0% of stand-alone coiling (OR 1.30, 95% CI 1.16–1.46). Likewise, recanalization rates of SAC were lower (4.8% vs. 16.6%, OR 0.28, 95% CI 0.16–0.50). The 6-month angiographic results in our series are within the range cited by Zhang et al.^11^. In particular, the superior immediate and 6-month aneurysm occlusion of SAC becomes evident in the adjusted analysis which accounts for the comparably complex aneurysm morphology of SAC-treated aneurysms. A large aneurysm size and neck width have been identified as risk factors for incomplete aneurysm occlusion and recanalization, both for stent-assisted coiling^23^ and stand-alone coiling^24^. A high packing density and immediate complete occlusion are well-known requisites to minimize the risk of recanalization^24–26^. Intracranial stents serve as scaffold for neo-endothelialisation and prevent protrusion of the implanted coils which facilitates a dense coil packing promoting progressive and durable aneurysm occlusion.

Taking into account both clinical and angiographic outcome, the results of the meta-analysis by Zhang et al. and our data indicate that SAC of large and wide-necked ruptured aneurysms is safe and effective, providing better aneurysm occlusion and similar clinical outcome to stand-alone coiling^11^. In line, Tähtinen et al. recommended explicitly the use of SAC for morphologically complex ruptured aneurysms^9^. A large prospective randomized clinical trial would be ultimately required to provide definitive information on the best therapeutic approach for endovascular treatment of ruptured intracranial aneurysms.

Besides conventional stents, novel stent-like neck bridging devices have been established for treatment of ruptured intracranial aneurysms. For the pCONus (Phenox, Bochum, Germany), a laser-cut stent with a distal crown which is placed inside the aneurysm, Aguilar Pérez et al. reported intraprocedural complications in 9.5% and 62% achieved a good functional outcome. The complete occlusion rate was 56% and the recurrence rate was 25%^27^. For the successor version, the pCONus HPC, the authors suggested the potential used of single antiplatelet therapy^28^. Other neck bridging devices, such as the endovascular clip system (eCLIPs, Evasc Medical Systems, Vancouver, Canada)^29^ and the PulseRider (Johnson & Johnson, New Brunswick, NJ, USA)^30^ showed an adequate safety and efficacy profile in preliminary studies, but have been mainly studied for unruptured aneurysms. These devices may represent a treatment alternative for selected aneurysms, especially for wide-necked and bifurcation aneurysms. For devices with a reduced metal surface, single antiplatelet therapy might suffice, which could further increase the safety of stent-assisted procedures.

The limitations of this study are mainly related to its retrospective design and the moderate number of included patients. Although the performed IPTW analysis enhances the validity of our results, we cannot exclude a potential selection bias. Moreover, clinical outcome was not determined systematically but retrospectively based on the neurological descriptions in the medical charts. The angiographic follow-up rate was only 56% (159/284), which may not reflect the real angiographic results. Furthermore, few patients treated by coiling alone underwent follow-up by CTA and MRA, which might impede imaging analysis. In this context, aneurysm occlusion was not determined by a core laboratory which might bias the interpretation of the angiographic results^31^. Finally, we did not report long-term outcome, however, it can be expected that the differences in aneurysm occlusion between SAC and stand-alone coiling might become more evident at long-term follow-up.

## Conclusions

The results of this IPTW-adjusted analysis indicate that SAC of ruptured intracranial aneurysms can provide improved angiographic results with no additional morbidity when compared to stand-alone coiling in carefully selected cases. Moreover, the results indicate that the risks of thromboembolic and hemorrhagic events are mainly related to the aneurysm morphology and not to the treatment modality. The introduction of novel devices with reduced metal surface and potential single antiplatelet therapy may further increase the safety of SAC in the future.

## Methods

The study protocol was approved by the local ethics committee of the University Hospital of Cologne (IRB 13-104). The need for informed consent was waived by the local ethics committee of the University Hospital of Cologne. The study was conducted in accordance with the STROBE guidelines in compliance with the national legislation and the Code of Ethical Principles for Medical Research Involving Human Subjects of the World Medical Association (Declaration of Helsinki).

### Inclusion and exclusion criteria

Consecutive SAH patients treated at a single center between January 2010 and December 2019 were retrospectively reviewed. All patients that underwent endovascular treatment for an acutely ruptured intracranial aneurysm within 14 days after ictus were considered. This time period was selected, because patients were surveilled at the intensive care unit for at least 14 days after ictus. Patients treated with coiling only, balloon-assisted coiling and stent-assisted coiling were included. Exclusion criteria were: (1) Microsurgical clipping, (2) flow-diverter implantation (3) treatment with the Woven Endobridge (WEB), (4) parent artery occlusion, (5) recurrent aneurysms, (6) partially thrombosed aneurysms, (7) fusiform aneurysms, and (8) dissecting aneurysms. Patients treated by coiling only and balloon-assisted coiling were subsumed in the “coiling” group.

### Procedure

All procedures were performed via a transfemoral approach with the patient under general anesthesia in a biplane angiosuite (Philips, Best, the Netherlands). Intracranial stenting was performed using a Headway 17 (Microvention, Tustin, CA, USA) or Prowler Select Plus (Codman Neurovascular, Raynham, MA, USA) microcatheter depending on the individual microstent type. The following stent types were employed: Acclino (Acandis, Pforzheim, Germany), Solitaire AB (Medtronic, Dublin, Ireland), Enterprise (Johnson & Johnson, New Brunswick, NJ, USA), Neuroform Atlas (Stryker, Kalamazoo, MI, USA), and LVIS jr (Microvention, Tustin, CA, USA). The adjunctive use of stents, stent type and number of stents was left to the discretion of the neurointerventionalist. The standard approach of SAC consisted of stent deployment across the aneurysm neck, followed by probing the aneurysm sac with a microcatheter through the stent interstices and final coil embolization. Furthermore, the microcatheter jailing technique was used, in which the stent is deployed after microcatheterization of the aneurysm sac by an additional microcatheter and before coil deployment. Bifurcation aneurysms were treated either with a single stent which is placed across the branching vessels or with two stents using the Y-stent technique.

In case of intra-procedural re-rupture, the rupture site was immediately embolized with coils to stop the bleeding. Therafter, the aneurysm was treated as originally planned. After the procedure, a CT scan was performed to determine the extent of re-hemorrhage. In case of intra-procedural thromboembolism, a loading dose of intra-arterial tirofiban (infusion rate: 0.4 µg/kg/min) was applied for 30 min. In some cases additional mechanical thrombectomy was performed. A control CT scan with perfusion sequences was performed to evaluate cerebral infarction.

Angiographic follow-up was performed 6 months after the procedure using DSA in the majority of cases. In few patients treated by coiling alone, magnetic resonance angiography and computed tomography angiography was used instead of DSA. The Raymond-Roy occlusion classification (RROC) was used to evaluate aneurysm occlusion: 1, complete occlusion, 2, neck remnant, and 3, aneurysm remnant. Aneurysm recurrence was defined as an increase of the RROC score at follow-up compared to post-treatment. Upon proof of aneurysm remnants, the need and modality for retreatment was discussed within an interdisciplinary neurovascular board.

### Anti-aggregation therapy

I.v. Heparin was not administered routinely. In case of adjunctive stent deployment, tirofiban (Aggrastat, Merck, West Point, PY, USA) was applied weight adapted according to the manufacturer`s guidelines, starting promptly before stent placement and continued for 16–24 h after the procedure. Tirofiban is given intravenously at an initial infusion rate of 0.4 µg/kg/min for 30 min. At the end of the initial infusion, tirofiban is continued at a maintenance infusion rate of 0.1 µg/kg/min. Thereafter, the patients were loaded with 500 mg acetylsalicylic acid (ASA) and 300 mg clopidogrel. Maintenance anti-platelet therapy consisted of ASS 100 mg/day life-long and clopidogrel 75 mg/day.

### Data collection and definition of outcome parameters

Medical charts and operation records were retrospectively reviewed to determine the following parameters: patient age, sex, World Federation of Neurosurgical Societies (WFNS) grading scale, Fisher scale, time interval between ictus and treatment, length of hospital stay, procedural adverse events, in-hospital mortality, external ventricular drain (EVD)/ventriculoperitoneal shunt placement and neurological status at follow-up. Procedural adverse events include intraoperative thromboembolic events (e.g. in-stent stenosis, thromboembolism) and intraoperative hemorrhagic events (e.g. aneurysm perforation, rebleeding) independently of their clinical significance. Native CT scans and CT angiography were reviewed in order to determine vasospasm and cerebral infarction. Procedure-related infarction was defined to be spatially and temporally related to the procedure without the occurrence of concomitant vasospasm. Furthermore, 30-day overall ischemic stroke rates (including procedure-related infarction and delayed cerebral ischemia) are reported. In patients that underwent EVD and/or VP-shunt placement, postoperative CT scans were reviewed to identify ventriculostomy-related hemorrhage, which was defined as a new intraparenchymal bleeding along the ventricular catheter tract. Functional outcome was assessed at 6-month follow-up determining the modified Rankin scale (mRS) score on the basis of the neurological status. Patients that were not available for 6-month follow-up visits were contacted by phone. A mRS score ≤ 2 was defined as favourable outcome and a mRS score > 2 as unfavourable outcome, whereby a mRS score of 6 defines death. Four-vessel digital subtraction angiography (DSA) scans were reviewed to determine aneurysm size, neck width, and dome-to-neck (D/N) ratio.

Favourable functional outcome at 6-month follow-up was defined as primary outcome measure. Secondary outcome measures were intraoperative thromboembolic and hemorrhagic events, immediate complete occlusion, procedure-related and overall cerebral infarction, in-hospital mortality, ventriculostomy-related haemorrhage, 6-month complete occlusion and retreatment.

### Statistical analysis

Qualitative data are presented as numbers and percentages. Groups are compared using the Chi-Square and the Fisher exact text, when appropriate. Quantitative data are presented as means and standard deviation and compared using the unpaired t-test and the Mann–Whitney U test. Normal distribution of quantitative parameters was evaluated with the Shapiro–Wilk test. Inverse probability of treatment weighting (IPTW) based on individual propensity scores was applied to counteract a potential selection bias and to control for differences in baseline characteristics between the coiling alone and the SAC group. This methods allows a retrospective randomization. An individual propensity score was calculated for each patient as the predicted probability for SAC by using a multivariate logistic regression model with stent-assisted treatment as the response and the following covariates: patient age, sex, WFNS grade, Fisher grade, aneurysm location, aneurysm size, and neck width. In the IPTW method, each patient was weighted by the inverse probability of being in either the coiling alone or the SAC group. This approach leads to the creation of two synthetic study groups which have similar propensity scores and thus comparable baseline characteristics. Statistical analysis was performed using SPSS software (IBM SPSS Statistics for Windows, Version 25.0, Armonk, NY, USA). A p-value < 0.05 was considered as statistically significant. For propensity score analysis, the R 3.3.0 plug-in (https://cran.r-project.org/) was installed.

## Data Availability

All data will be made available upon request in an anonymized manner.
